# KCNK9 mediates the inhibitory effects of genistein on hepatic metastasis from colon cancer

**DOI:** 10.1016/j.clinsp.2022.100141

**Published:** 2023-03-09

**Authors:** Yuan Cheng, Yi Tang, Yiming Tan, Juan Li, Xuping Zhang

**Affiliations:** a Department of Pharmacology Laboratory, Hospital of Chengdu University of Traditional Chinese Medicine, Chengdu, China; b Department of Pharmacy, Chengdu Second People's Hospital, Chengdu, China

**Keywords:** KCNK9, Genistein, Colon cancer, Metastasis

## Abstract

**Objective:**

The tyrosine-protein kinase inhibitor, genistein, can inhibit cell malignant transformation and has an antitumor effect on various types of cancer. It has been shown that both genistein and KNCK9 can inhibit colon cancer. This research aimed to investigate the suppressive effects of genistein on colon cancer cells and the association between the application of genistein and KCNK9 expression level.

**Methods:**

The Cancer Genome Atlas (TCGA) database was used to study the correlation between the KCNK9 expression level and the prognosis of colon cancer patients. HT29 and SW480 colon cancer cell lines were cultured to examine the inhibitory effects of KCNK9 and genistein on colon cancer *in vitro*, and a mouse model of colon cancer with liver metastasis was established to verify the inhibitory effect of genistein *in vivo*.

**Results:**

KCNK9 was overexpressed in colon cancer cells and was associated with a shorter Overall Survival (OS), a shorter Disease-Specific Survival (DFS), and a shorter Progression-Free Interval (PFI) of colon cancer patients. *In vitro* experiments showed that downregulation of KCNK9 or genistein application could suppress cell proliferation, migration, and invasion abilities, induce cell cycle quiescence, promote cell apoptosis, and reduce epithelial-mesenchymal transition of the colon cancer cell line. *In vivo* experiments revealed that silencing of KCNK9 or application of genistein could inhibit hepatic metastasis from colon cancer. Additionally, genistein could inhibit KCNK9 expression, thereby attenuating Wnt/β-catenin signaling pathway.

**Conclusion:**

Genistein inhibited the occurrence and progression of colon cancer through Wnt/β-catenin signaling pathway that could be mediated by KCNK9.

## Highlights

• High KCNK9 expression level is correlated with colon cancer patients' poor survival.
• KCNK9 promotes proliferation, migration, invasion, EMT, and Wnt signaling pathway.
• KCNK9 mediates the inhibitory effects of genistein on colon cancer liver metastasis.

## Introduction

Colorectal cancer is the second most prevalent cancer in men and the third in women worldwide.^1^ Around 1.8 million new cases of colon cancer are annually diagnosed, accounting for about 10% of the total number of new cases diagnosed; moreover, approximately 900,000 people die of colon cancer every year (≈9% of all cancer-related deaths).^2^ Colon adenocarcinoma is the most frequent pathological type of colon cancer. The treatment for colon cancer patients was still limited, although molecular targeted agents and immunotherapies have been largely developed.^3^ In clinical practice, patients with stage I or II colon cancer have mainly received partial or total colectomy alone; while about two-thirds of those with stage III disease have received neoadjuvant chemotherapy plus colectomy to lower the risk of colon cancer recurrence.^3^ Hence, it is of vital importance to find out new drugs and develop new therapeutic targets for colon cancer.

KCNK9 is an important member of the two-pore domain potassium (K2P) channel family, which preserves cell resting membrane potential and adjusts action potential discharge.^4^ KCNK9 is associated with several types of human malignant tumors, owing to its amplification status in human tumors and its capacity to promote neoplasm development.^5^, ^6^, ^7^, ^8^ It has been shown that the potassium channel activity of KCNK9 was directly associated with its cancer-promoting function.^9^ The prognostic and diagnostic capabilities of KCNK9 for Hepatocellular Carcinoma (HCC) have been examined.^7^ However, the underlying mechanism that how KCNK9 exerts its cancer-promoting function remains to be further studied and verified.

Genistein is a type of natural isoflavone with a wide range of pharmacological properties and cellular regulation capacities.^10^ Genistein has been shown to modulate various pathways related to metabolic disorders and cancer.^11^^,^^12^ The molecular mechanisms that genistein exerts its influence on tumorigenesis include suppression of cell replication and inflammation, as well as regulation of epigenetic modifications.^11^^,^^13^^,^^14^ Genistein derivatives have been shown to inhibit phosphorylation of Epidermal Growth Factor Receptor (EGFR) at two tyrosine sites in a concentration-dependent manner after 24h of treatment in two colon cancer cell lines.^15^ One study showed that genistein could reduce K2p9.1 (KCNK9) current using Xenopus oocytes and Chinese hamster ovary cells.^16^ Previous studies have shown that genistein could stimulate FOXO3 to restrain EGF-induced proliferation in colon cancer cells by targeting the PI3K/Akt signaling pathway.^17^ Apart from that, genistein attenuated Wnt signaling pathway by upregulating the expression of microRNA-574-3p in prostate carcinoma cells^18^ and downregulating the expression of onco-miR-1260b in renal cancer cells.^19^ Genistein has been used in different clinical trials for cancer and metabolic diseases.^20^, ^21^, ^22^, ^23^ A phase I/II pilot study revealed that the addition of genistein to chemotherapy was safe and tolerable for the treatment of metastatic colorectal cancer.^22^ Several studies have concentrated on the functions of genistein in colon cancer, and have reported the involvement of genistein in a variety of signaling pathways and regulatory mechanisms. However, the present research team found a new regulatory mechanism of genistein in colon cancer. In the present study, the authors provided new evidence that genistein could inhibit cell replication and induce apoptosis of colon cancer cells by attenuating the Wnt/β-catenin signaling pathway, which could be mediated by KCNK9. This study revealed a new anti-tumor effect of genistein on colon carcinoma.

## Materials and methods

### Human colon tissues

A total of 4 specimens of patients with colon adenocarcinoma were collected from the Hospital of Chengdu University of Traditional Chinese Medicine (Chengdu, China). Fresh colon adenocarcinoma tissues and their corresponding adjacent normal tissues were collected after surgery and put into liquid nitrogen for preservation. All patients signed a written informed consent form before surgery. The study was approved by the Ethics Committee of the Hospital of Chengdu University of Traditional Chinese Medicine (Approval n° 2021DL-006).

### A mouse model of colon cancer with liver metastasis

A total of 24 male nude mice (age, 4‒6-week-old) were bred in Specific Pathogen-Free (SPF) cages, with ventilated air and adequate food and water. In order to establish a mouse model of colon cancer with liver metastasis, 1 × 10^6^ colon cancer cells were injected into the spleen tissues of mice. After 6-weeks of cultivation, mice were sacrificed by intraperitoneal injection of 200 mg/kg pentobarbital sodium, and the number and dimensions of liver metastasis were recorded and assessed. All procedures regarding animals in this study follow the recommendations in the ARRIVE guidelines.

### Cell culture

Human colon cancer cell lines (HT29 and SW480) and a normal colon epithelial cell line (FHC) were purchased from the European Collection of Authenticated Cell Cultures (ECACC; Salisbury, UK). These three cell lines were cultured in a Roswell Park Memorial Institute (RPMI)-1640 medium (#11875093; Gibco, New York, NY, USA), containing 10% fetal bovine serum (FBS; #10099-141; Gibco), penicillin (100 U/mL) and streptomycin (100 μg/mL) (#C14-15140-122; Gibco), and incubated at 37°C under 5% CO_2_ and 95% humidity. Knocked-down cell lines of KCNK9 in HT29 and SW480 were established by shRNA, and their sequences are listed in Supplementary Table 1.

### Cell transfection

Cells were resuspended in culture media and seeded into 24-well plates on the day before transfection. Then, 1 μg (50 pmoL) shRNA was diluted by 25 μL OPTI-MEM, and 1 μL lipofectamine was diluted by 25 μL OPTI-MEM. The two diluted reagents were mixed in a 1:1 ratio and incubated at room temperature for 15 min. The DNA-lipid complex was administered to cells using complete culture media. The media were refreshed after 6h and the transfected cells were cultured at 37°C for 2‒4 days before analysis.

### Cell proliferation assay (Cell Counting Kit-8 [CCK-8] assay)

Two thousand cells for each well were resuspended in culture media and seeded into 96-well plates. Cell-Counting Kit 8 (Dojindo, #ck-04, Japan) was used to determine the cell proliferation rate according to the manufacturer's instructions at 24h, 48h, and 72h after cell adherence. A microplate reader was applied to measure the absorbance.

### Plate clone formation assay

In this study, 2 × 10^3^ cells were resuspended with an FBS-free medium and seeded into each well of 6-well plates. After a two-week incubation, all cells were fixed with 4% paraformaldehyde solution for 15 min and stained with crystal violet for 30 min at room temperature. After being washed twice with Phosphate-Buffered Saline (PBS) solution, the number of cells in each well was counted.

### Wound healing assay

Cells were seeded into a 12-well plate with a 95% confluence in monolayer. After attachment, a 10 μL plastic pipette tip was used to make a scratch along the bottom of the plate. Cells were washed for several times with culture media to remove residual cells. The wounds were then photographed after 24h.

### Cell invasion assay

Herein, 10^5^ cells were resuspended in serum-free 1640 media and then administered into the upper chamber. The lower containers were filled with complete growth media, containing 10% FBS. The cell invasion assay was performed using BioCoat Matrigel Invasion chambers (#354480; BD Biosciences, San Diego, CA, USA), according to the manufacturer's instructions.

### Quantitative real-time polymerase chain reaction (RT-qPCR)

TRIzol reagent (#15596026; Invitrogen, Carlsbad, CA, USA) was applied to isolate the total RNA of the cells and tissues, and its concentration was quantified by a NanoDrop™ 2000 spectrophotometer. The cDNA was produced by the reverse transcription kit (#RR036A; Takara, Shiga, Japan), according to the manufacturer's protocols. The StepOne Plus system (#4376600; Applied Biosystems, Waltham, MA, USA) was used to perform RT-qPCR. The primers used for RT-qPCR are listed in Supplementary Table 2.

### Western blotting

Total proteins of cells were obtained using RIPA lysis buffer (#89900; Thermo Fisher Scientific, Waltham, MA, USA) along with protease inhibitor cocktail and phosphorylase inhibitor. The concentration of the protein was quantified using the Bicinchoninic Acid (BCA) method. In detail, 25 μg protein of each sample was loaded onto a gel for separation and transferred to Polyvinylidene Difluoride (PVDF) membranes for exposure. Antibodies used in this experiment were listed as follows: anti-KCNK9 (#ab85289; Abcam, Cambridge, UK), anti-KCNK3/TASK1 (#ab135883; Abcam), anti-GAPDH (#ab8245; Abcam), anti-Ki67 (#ab16667; Abcam), anti-E-cadherin (#3195; Cell Signaling Technology [CST], Inc., Danvers, MA, USA), anti-N-cadherin (#13116; CST Inc.), anti-Bax (#5023; CST Inc.), anti-Bcl-2 (#15071; CST Inc.), anti-caspase3 (#9662; CST Inc.), anti-cleaved caspase-3 (#9654; CST Inc.), anti-PARP (#9532; CST Inc.), anti-cleaved PARP (#9185; CST Inc.), anti-beta catenin (#ab32572; Abcam), anti-c-Myc (#ab185656; Abcam), anti-P53 (#ab26; Abcam), anti-cyclin D1 (#ab16663; Abcam), anti-CDK6 (#ab124821; Abcam), anti-CDK4 (#ab108357; Abcam), anti-vimentin (#ab92547; Abcam), and anti-vimentin (#ab27568; Abcam).

### Hematoxylin & Eosin (H&E) staining

Paraffin-embedded tissue sections were dewaxed with xylene for 10 min each time and then soaked in absolute ethanol, 90% ethanol, 80% ethanol, and 70% ethanol for five minutes, respectively. After dewaxed, the tissue sections were rinsed with running water for 5-min. The tissue sections were then stained with hematoxylin dye. Then, 1% hydrochloric acid alcohol was used for differentiation. Eosin dye was applied for 3-min after being washed with water; 95% ethanol, absolute ethanol, and xylene were used twice successively for 5-min for dehydration.

### Immunohistochemistry (IHC)

IHC was performed according to the standard protocol. Briefly speaking, tissues were fixed with formalin and embedded into paraffin, and the tissue sections were then dewaxed and rehydrated. The antigen was retrieved by a citric acid buffer. Thereafter, the tissue sections were incubated with primary antibodies and stained with 3, 3′-Diaminobenzidine (DAB).

### The Cancer Genome Atlas (TCGA) transcriptome data analysis

The transcriptome data of colon cancer obtained from the TCGA database were analyzed using “TCGAbiolinks” R package, and the differential expression analysis of genes was carried out via “DESeq2” R package.

### Gene set enrichment analysis (GSEA)

To illustrate the biological correlations of the obtained gene expression profiles, the transcriptome data were compared using GSEA (http://www.gsea-msigdb.org/gsea/). GSEA used the weighted Kolmogorov-Smirnov method to determine whether the distribution of genes in the gene set is different from the normal distribution. False Discovery Rate (FDR) < 0.05 and adjusted p-value < 0.05 were considered statistically significant.

### Statistical analysis

All data were displayed in the form of mean ± standard deviation and analyzed by two-sided Student's *t*-test or one- or two-way analysis of variance (ANOVA) after three independent experiments. When the data did not conform to the normal distribution and homogeneity of variance, the nonparametric test was applied. The log-rank test and Cox regression model were used in the survival analysis. GraphPad Prism 9.0 software (GraphPad Software Inc., San Diego, CA, USA) and R software-related packages were used to perform the statistical analysis; p < 0.05 was considered statistically significant (* p < 0.05; ** p < 0.01; *** p < 0.001).

## Results

### KCNK9 expression level was upregulated in colon adenocarcinoma cells

**Figure 1:**
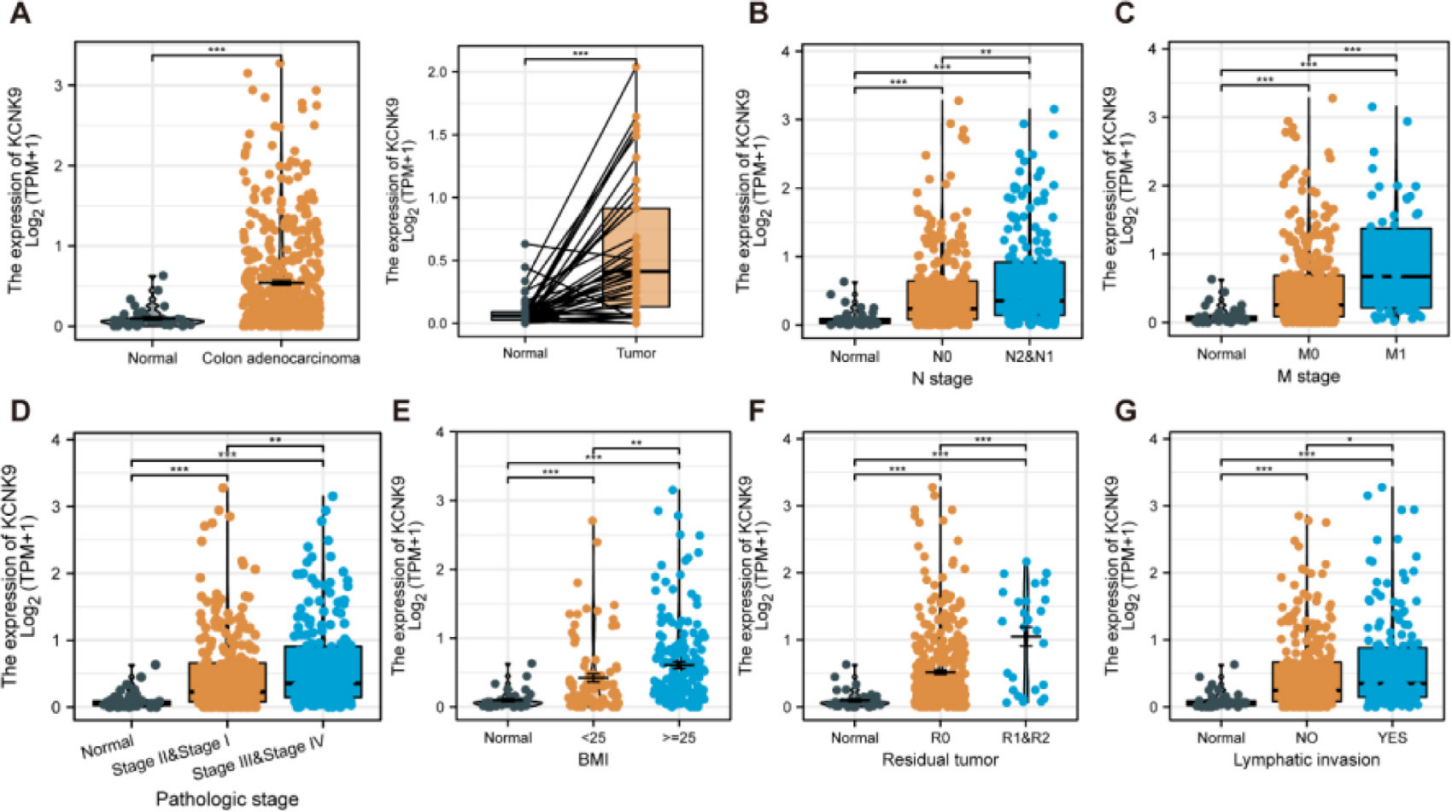
KCNK9 expression in different subgroups of patients with colon cancer in TCGA COAD dataset. (A) The KCNK9 expression level was higher in tumoral tissues than that in normal tissues (p < 0.001). (B) The KCNK9 expression level was upregulated in higher N stages (normal vs. N0, p < 0.001; normal vs. N1 & N2, p < 0.001; N0 vs. N1 & N2, p < 0.01). (C) KCNK9 was upregulated in higher M stages (normal vs. M0, p < 0.001; normal vs. M1, p < 0.001; M0 vs. M1, p < 0.001). (D) The KCNK9 expression level was higher in stages III and IV tumor tissues than that in stages I and II tissues (normal vs. stages I & II, p < 0.001; normal vs. stages III & IV, p < 0.001; stages I & II vs. stages III & IV, p < 0.001). (E) KCNK9 was overexpressed in the higher BMI score subgroup (normal vs. BMI < 25 kg/m^2^, p < 0.001; normal vs. BMI ≥ 25 kg/m^2^, p < 0.001; BMI < 25 vs. BMI ≥ 25 kg/m^2^, p < 0.01). (F) KCNK9 was overexpressed in residual tumors (normal vs. R0, p < 0.001; normal vs. R1 & R2, p < 0.001; R0 vs. R1 & R2, p < 0.001). (G) KCNK9 was overexpressed in tumors with lymphatic invasion (normal vs. lymphatic invasion, p < 0.001; normal vs. non-lymphatic invasion, p < 0.001; lymphatic invasion vs. non-lymphatic invasion, p < 0.05). p-values were calculated using the Student's *t*-test.

In order to determine KCNK9 expression in colon adenocarcinoma cells, the authors first analyzed the expression data of KCNK9 using the TCGA database. The results showed that KCNK9 expression was upregulated in colon adenocarcinoma cells (Fig. 1A). Further analysis of the correlation between TNM staging and KCNK9 expression revealed that the KCNK9 expression level was elevated gradually with the increase of N or M stage (Figs. 1B, C). Then, KCNK9 expression was analyzed in tumors with different pathological stages and patients with different Body Mass Index (BMI) values. It was found that tumors with advanced stages (stages III and IV) had a higher KCNK9 expression level than that of lower stages (stages I and II) or normal tissues (Fig. 1D). KCNK9 was overexpressed in patients with a higher BMI value (Fig. 1E). In addition, KCNK9 expression level in residual tumors and lymphatic invasion loci was analyzed. The KCNK9 expression level in patients with R1 and R2 resection was higher than that in patients with R0 resection or in normal tissues (Fig. 1F). The KCNK9 expression level in the lymphatic invasion group was higher than that in the non-lymphatic invasion group and higher than that in normal tissues (Fig. 1G). The authors further examined the relationship between KCNK9 expression level and clinicopathologic characteristics of colon adenocarcinoma patients. Patients were divided into a high KCNK9 expression group (n = 239) and a low KCNK expression group (n = 239) according to the KCNK9 expression level. The results revealed that KCNK9 expression level was correlated with tumor N stage, M stage, and pathological stage, and was also associated with residual tumors and a history of colon polyps (Table 1).

**Table 1:** Correlation between KCNK9 expression and clinicopathologic characteristics of colon adenocarcinoma patients.

Characteristics	Low KCNK9 expression	High KCNK9 expression	p
N	239	239	
T stage, n (%)			0.533
T1	7 (1.5%)	4 (0.8%)	
T2	45 (9.4%)	38 (8%)	
T3	155 (32.5%)	168 (35.2%)	
T4	32 (6.7%)	28 (5.9%)	
N stage, n (%)			**0.013**
N0	157 (32.8%)	127 (26.6%)	
N1	49 (10.3%)	59 (12.3%)	
N2	33 (6.9%)	53 (11.1%)	
M stage, n (%)			**0.004**
M0	182 (43.9%)	167 (40.2%)	
M1	21 (5.1%)	45 (10.8%)	
Pathological stage, n (%)			**0.004**
Stage I	45 (9.6%)	36 (7.7%)	
Stage II	106 (22.7%)	81 (17.3%)	
Stage III	64 (13.7%)	69 (14.8%)	
Stage IV	21 (4.5%)	45 (9.6%)	
Primary therapeutic outcomes, n (%)			0.213
PD	9 (3.6%)	16 (6.4%)	
SD	1 (0.4%)	3 (1.2%)	
PR	9 (3.6%)	4 (1.6%)	
CR	102 (40.8%)	106 (42.4%)	
Gender, n (%)			1.000
Female	113 (23.6%)	113 (23.6%)	
Male	126 (26.4%)	126 (26.4%)	
Race, n (%)			0.487
Asian	4 (1.3%)	7 (2.3%)	
Black or African American	34 (11.1%)	29 (9.5%)	
White	111 (36.3%)	121 (39.5%)	
Residual tumor, n (%)			**0.042**
R0	179 (47.9%)	167 (44.7%)	
R1	1 (0.3%)	3 (0.8%)	
R2	7 (1.9%)	17 (4.5%)	
CEA level, n (%)			0.092
≤ 5	100 (33%)	96 (31.7%)	
> 5	43 (14.2%)	64 (21.1%)	
Perineural invasion, n (%)			0.899
No	68 (37.6%)	67 (37%)	
Yes	22 (12.2%)	24 (13.3%)	
Lymphatic invasion, n (%)			0.050
No	141 (32.5%)	125 (28.8%)	
Yes	72 (16.6%)	96 (22.1%)	
History of colon polyps, n (%)			**0.026**
No	121 (29.7%)	141 (34.6%)	
Yes	85 (20.8%)	61 (15%)	
Colon polyps present, n (%)			0.704
No	80 (32.1%)	82 (32.9%)	
Yes	40 (16.1%)	47 (18.9%)	
Neoplasm type, n (%)			1.000
Colon adenocarcinoma	239 (50%)	239 (50%)	
Rectum adenocarcinoma	0 (0%)	0 (0%)	
Age, median (IQR)	69 (60, 79)	69 (58, 76.5)	0.345
Height, median (IQR)	167.8 (160, 175)	170.05 (163, 176)	0.065
Weight, median (IQR)	80.2 (64.1, 97.5)	81 (67.08, 91.03)	0.623
BMI, median (IQR)	27.22 (23.81, 34.94)	27.1 (24.03, 31.71)	0.642

### High KCNK9 expression level associated with a poorer prognosis of patients with colon cancer

**Figure 2:**
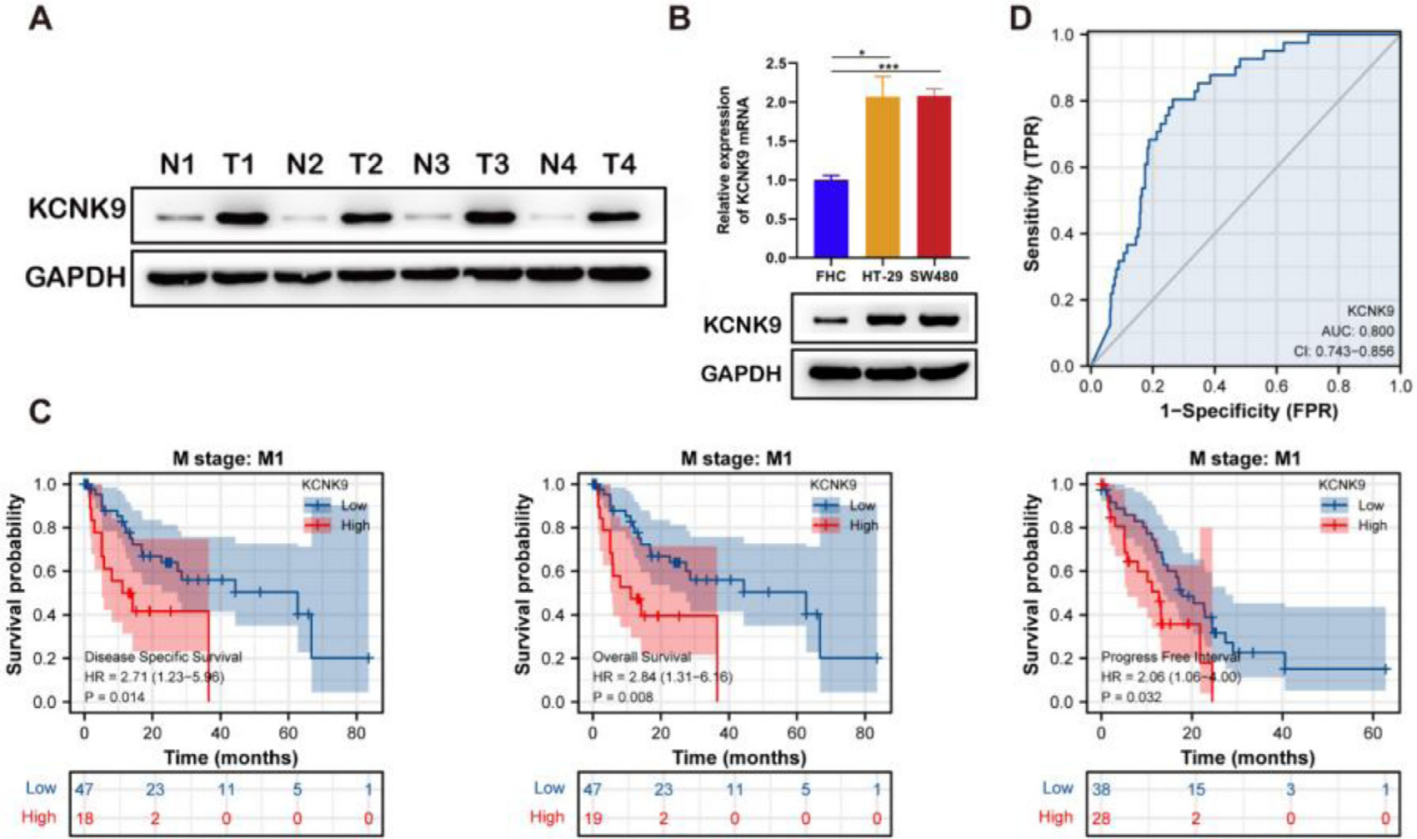
KCNK9 expression was upregulated in colon cancer and was associated with patients’ survival. (A) KCNK9 was overexpressed in tumor tissues than in normal tissues. (B) The mRNA and protein levels of KCNK9 in colon cancer cell lines (HT-29 and SW480) and the normal colon epithelial cell line (FHC) were examined by RT-qPCR and Western blotting. KCNK9 was overexpressed in colon cancer cell lines (p < 0.001); data were presented as mean ± Standard Deviation (SD); p-values were calculated using the Student's *t*-test. (C) Kaplan-Meier method was used to evaluate the overall survival in TCGA COAD according to KCNK9 expression level; (D) ROC curve of KCNK9 in TCGA COAD.

Western blotting was applied to detect KCNK9 expression in colon cancer tissues and cells. KCNK9 expression was upregulated in tumor tissues at the protein level (Fig. 2A). Next, KCNK9 expression was identified in normal colon epithelial cell line (FHC) and two colon cancer cell lines, HT-29 and SW480, by RT-qPCR and Western blotting. Compared with normal colon epithelial cells, KCNK9 expression was also upregulated in colon cancer cell lines (Fig. 2B). In order to further explore the relationship between KCNK9 expression and the prognosis of patients with colon cancer, the survival curve of patients with tumor M1 stage in the TCGA COAD cohort was analyzed using Kaplan-Meier method. 66 patients were divided into a high KCNK9 expression group and a low KCNK9 expression group according to the KCNK9 expression level. The results showed that the Disease-Specific Survival (DSS) and Overall Survival (OS) of patients in the high KCNK9 expression group were higher than those in the low KCNK9 expression group. Moreover, the Progression-Free Interval (PFI) in the low KCNK9 expression group was longer than that in the high KCNK9 expression group (Fig. 2C). Receiver Operating Characteristic (ROC) curve analysis showed that KCNK9 expression level could be used as a predictive marker for colon cancer. The Area Under the Curve (AUC) was 0.800 (95% Confidence Interval [95% CI 0.743‒0.856]) (Fig. 2D). Univariate Cox regression analysis found that T3 & T4 stage, N1 stage, N2 stage, M1 stage, III & IV pathological stage, lymphatic invasion, tumor complete response after primary therapy, age > 65 years old, BMI ≥ 25 kg/m^2^, R1 & R2 residual tumor, carcinoembryonic antigen (CEA) > 5 ng/mL, and KCNK9 expression level was associated with colon cancer patients’ OS (Table 2). Apart from OS, univariate Cox regression analysis showed that T3 & T4 stage, N1 stage, N2 stage, M1 stage, III & IV pathological stage, lymphatic invasion, perineural invasion, tumor complete response after primary therapy, R1 & R2 residual tumor, CEA > 5 ng/mL, and KCNK9 expression were correlated with DSS (Table 3). In addition, univariate Cox regression analysis of PFI revealed that T3 & T4 stage, N2 stage, M1 stage, III & IV pathological stage, lymphatic invasion, perineural invasion, tumor partial response, and complete response after primary therapy, R1 & R2 residual tumor, CEA > 5 ng/mL, and KCNK9 expression were relevant to PFI (Table 4).

**Table 2:** Univariate Cox regression analysis of clinical characteristics associated with overall survival.

Characteristics	Total (N)	Hazard ratio (95% CI)	p
T stage	476		
T1 & T2	94	Reference	
T3 & T4	382	3.072 (1.423‒6.631)	**0.004**
N stage	477		
N0	283	Reference	
N1	108	1.681 (1.019‒2.771)	**0.042**
N2	86	4.051 (2.593‒6.329)	**< 0.001**
M stage	414		
M0	348	Reference	
M1	66	4.193 (2.683‒6.554)	**< 0.001**
Pathological stage	466		
I & II stage	267	Reference	
III & IV stage	199	2.947 (1.942‒4.471)	**< 0.001**
Lymphatic invasion	433		
No	265	Reference	
Yes	168	2.450 (1.614‒3.720)	**< 0.001**
Perineural invasion	181		
No	135	Reference	
Yes	46	1.940 (0.982‒3.832)	0.056
Primary therapeutic outcomes	250		
PD	25	Reference	
SD	4	0.930 (0.120‒7.183)	0.944
PR	13	0.271 (0.062‒1.191)	0.084
CR	208	0.087 (0.044‒0.173)	**< 0.001**
Gender	477		
Female	226	Reference	
Male	251	1.101 (0.746‒1.625)	0.627
Race	306		
Asian	11	Reference	
Black or African American	63	0.927 (0.208‒4.133)	0.921
White	232	0.810 (0.196‒3.346)	0.771
Age (years old)	477		
≤ 65	194	Reference	
> 65	283	1.610 (1.052‒2.463)	**0.028**
Weight (kg)	273		
≤ 90	189	Reference	
> 90	84	0.601 (0.292‒1.238)	0.167
Height (cm)	256		
< 170	127	Reference	
≥ 170	129	0.786 (0.445‒1.389)	0.407
BMI (kg/m^2^)	256		
< 25	87	Reference	
≥ 25	169	0.549 (0.311‒0.969)	**0.038**
Residual tumor	373		
R0	345	Reference	
R1 & R2	28	4.364 (2.401‒7.930)	**< 0.001**
CEA level	302		
≤ 5	195	Reference	
> 5	107	3.128 (1.788‒5.471)	**< 0.001**
History of colon polyps	407		
No	262	Reference	
Yes	145	0.741 (0.442‒1.242)	0.255
Existence of colon polyps	249		
No	162	Reference	
Yes	87	1.324 (0.738‒2.373)	0.346
KCNK9	477	1.375 (1.052‒1.797)	**0.020**

**Table 3:** Univariate Cox regression analysis of clinical characteristics associated with disease-specific survival.

Characteristics	Total (N)	Hazard ratio (95% CI)	p
T stage	460		
T1 & T2	93	Reference	
T3 & T4	367	7.758 (1.896‒31.745)	**0.004**
N stage	461		
N0	275	Reference	
N1	105	2.601 (1.353‒5.000)	**0.004**
N2	81	6.357 (3.512‒11.504)	**< 0.001**
M stage	399		
M0	334	Reference	
M1	65	7.833 (4.597‒13.346)	**< 0.001**
Pathological stage	451		
I & II stage	259	Reference	
III & IV stage	192	6.085 (3.235‒11.447)	**< 0.001**
Lymphatic invasion	422		
No	255	Reference	
Yes	167	4.133 (2.361‒7.235)	**< 0.001**
Perineural invasion	180		
No	134	Reference	
Yes	46	2.977 (1.325‒6.686)	**0.008**
Primary therapeutic outcomes	249		
PD	24	Reference	
SD	4	0.000 (0.000‒Inf)	0.998
PR	13	0.241 (0.055‒1.060)	0.060
CR	208	0.032 (0.012‒0.082)	**< 0.001**
Gender	461		
Female	220	Reference	
Male	241	1.142 (0.697‒1.871)	0.599
Race	290		
Asian	11	Reference	
Black or African American	63	1.439 (0.184‒11.255)	0.729
White	216	0.702 (0.094‒5.263)	0.731
Age (years old)	461		
≤ 65	191	Reference	
> 65	270	1.165 (0.702‒1.933)	0.555
Weight (kg)	258		
≤ 90	174	Reference	
> 90	84	0.907 (0.380‒2.162)	0.825
Height (cm)	241		
< 170	122	Reference	
≥ 170	119	0.658 (0.284‒1.525)	0.329
BMI (kg/m^2^)	241		
< 25	79	Reference	
≥ 25	162	0.979 (0.415‒2.310)	0.961
Residual tumor	373		
R0	345	Reference	
R1 & R2	28	6.107 (3.225‒11.563)	**< 0.001**
CEA level	301		
≤ 5	194	Reference	
> 5	107	3.018 (1.543‒5.901)	**0.001**
History of colon polyps	396		
No	253	Reference	
Yes	143	0.907 (0.497‒1.657)	0.752
Existence of colon polyps	243		
No	161	Reference	
Yes	82	1.397 (0.648‒3.011)	0.393
KCNK9	461	1.518 (1.105‒2.086)	**0.010**

**Table 4:** Univariate Cox regression analysis of clinical characteristics associated with progression-free interval.

Characteristics	Total (N)	Hazard ratio (95% CI)	p
T stage	476		
T1 & T2	94	Reference	
T3 & T4	382	3.111 (1.631‒5.936)	**< 0.001**
N stage	477		
N0	283	Reference	
N1	108	1.564 (0.993‒2.465)	0.054
N2	86	4.761 (3.188‒7.111)	**< 0.001**
M stage	414		
M0	348	Reference	
M1	66	5.811 (3.921‒8.611)	**< 0.001**
Pathological stage	466		
I & II stage	267	Reference	
III & IV stage	199	3.061 (2.120‒4.419)	**< 0.001**
Lymphatic invasion	433		
No	265	Reference	
Yes	168	2.433 (1.679‒3.525)	**< 0.001**
Perineural invasion	181		
No	135	Reference	
Yes	46	2.362 (1.279‒4.363)	**0.006**
Primary therapeutic outcomes	250		
PD	25	Reference	
SD	4	0.815 (0.108‒6.163)	0.843
PR	13	0.321 (0.110‒0.941)	**0.038**
CR	208	0.085 (0.048‒0.152)	**< 0.001**
Gender	477		
Female	226	Reference	
Male	251	1.166 (0.822‒1.656)	0.389
Race	306		
Asian	11	Reference	
Black or African American	63	0.835 (0.247‒2.824)	0.772
White	232	0.530 (0.164‒1.711)	0.289
Age (years old)	477		
≤ 65	194	Reference	
> 65	283	0.975 (0.683‒1.391)	0.888
Weight (kg)	273		
≤ 90	189	Reference	
> 90	84	0.950 (0.568‒1.589)	0.845
Height (cm)	256		
< 170	127	Reference	
≥ 170	129	1.198 (0.741‒1.939)	0.461
BMI (kg/m^2^)	256		
< 25	87	Reference	
≥ 25	169	1.186 (0.709‒1.986)	0.515
Residual tumor	373		
R0	345	Reference	
R1 & R2	28	4.343 (2.554‒7.385)	**< 0.001**
CEA level	302		
≤ 5	195	Reference	
> 5	107	2.900 (1.844‒4.561)	**< 0.001**
History of colon polyps	407		
No	262	Reference	
Yes	145	0.744 (0.482‒1.150)	0.183
Existence of colon polyps	249		
No	162	Reference	
Yes	87	0.996 (0.607‒1.634)	0.987
KCNK9	477	1.296 (1.017‒1.650)	**0.036**

### Knockdown of KCNK9 inhibited the malignant phenotype of colon cancer cell lines (HT-29 and SW480)

**Figure 3:**
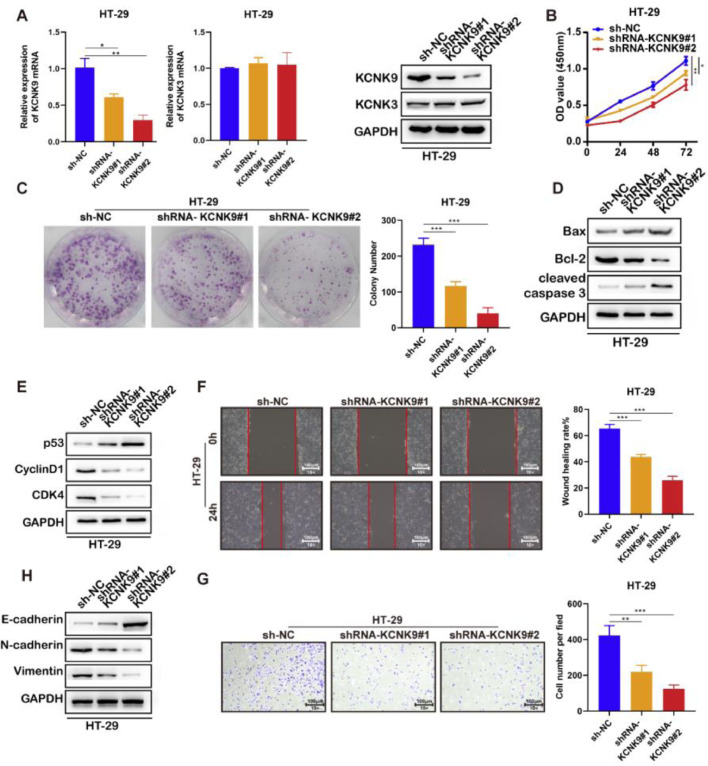
Knockdown of KCNK9 inhibited the malignant phenotype of colon cancer cell line HT-29. (A) KCNK9 expression was downregulated and KCNK3 remained unchanged after shRNA application; data were presented as mean ± Standard Deviation (SD); p-values were calculated using the Student's *t*-test. (B) CCK-8 assay showed decreased cell viability after silencing of KCNK9; data were presented as mean ± SD; p-values were calculated using the Student's *t*-test. (C) Colony formation assay showed reduced cell proliferation after silencing of KCNK9; data were presented as mean ± SD; p-values were calculated using the Student's *t*-test. (D) Expression levels of Bax, Bcl-2, caspase-3, cleaved caspase-3, PARP, and cleaved PARP were altered after silencing of KCNK9. (E) Expression levels of p53, CyclinD1, CDK6, and CDK4 were altered after silencing of KCNK9. (F) Wound healing assay showed decreased migration ability after silencing of KCNK9; data were presented as mean ± SD; p-values were calculated using the Student's *t*-test. (G) Transwell assay revealed declined cell invasion after silencing of KCNK9; data were presented as mean ± SD; p-values were calculated using the Student's *t*-test. (H) Expression levels of E-cadherin, N-cadherin, and vimentin were altered after silencing of KCNK9.

In order to further explore the role of KCNK9 expression in colon cancer, cells were transfected with short hairpin RNA (shRNA) to interfere with the KCNK9 expression level. Firstly, two KCNK9 knockdown cell lines of HT-29 (shrna-KCNK9#1 and shRNA-KCNK9#2) and their negative control cell line (Sh-NC) were constructed. The knockdown efficiency on the mRNA and protein expression levels of KCNK9 by shRNA was verified using RT-qPCR and Western blotting (Fig. 3A). CCK-8 assay was used to detect the cell viability of three different cell lines, and the results showed that knockdown of KCNK9 could inhibit the proliferation of HT-29 cells, and cells with a lower KCNK9 expression level had a lower cell viability (Fig. 3B). The outcome of colony formation assays also suggested that the proliferation ability of HT-29 cells decreased after the knockdown of KCNK9 (Fig. 3C). The authors further detected the expression levels of some apoptosis-related key proteins, such as Bax, Bcl-2, caspase-3, cleaved caspase-3, poly (ADP-ribose) Polymerase (PARP), and cleaved PARP. It was revealed that the knockdown of KCNK9 could induce cell apoptosis (Fig. 3D). Next, the expression levels of cell cycle-related proteins, including p53, CyclinD1, CDK6, and CDK4 were detected, and the results indicated that the knockdown of KCNK9 could induce the cell cycle quiescence (Fig. 3E). Wound healing assay showed that the inhibition of KCNK9 could restrain the migration capacity of colon cancer cells (Fig. 3F). The Epithelial-Mesenchymal Transition (EMT) level was examined by measuring the expression levels of E-cadherin, N-cadherin, and relevant proteins (e.g., vimentin). These findings showed that the knockdown of KCNK9 could suppress the EMT of colon cancer (Fig. 3H). Cell invasion assay revealed that the knockdown of KCNK9 decreased the invasion ability of colon cancer cells (Fig. 3G).

**Figure 4:**
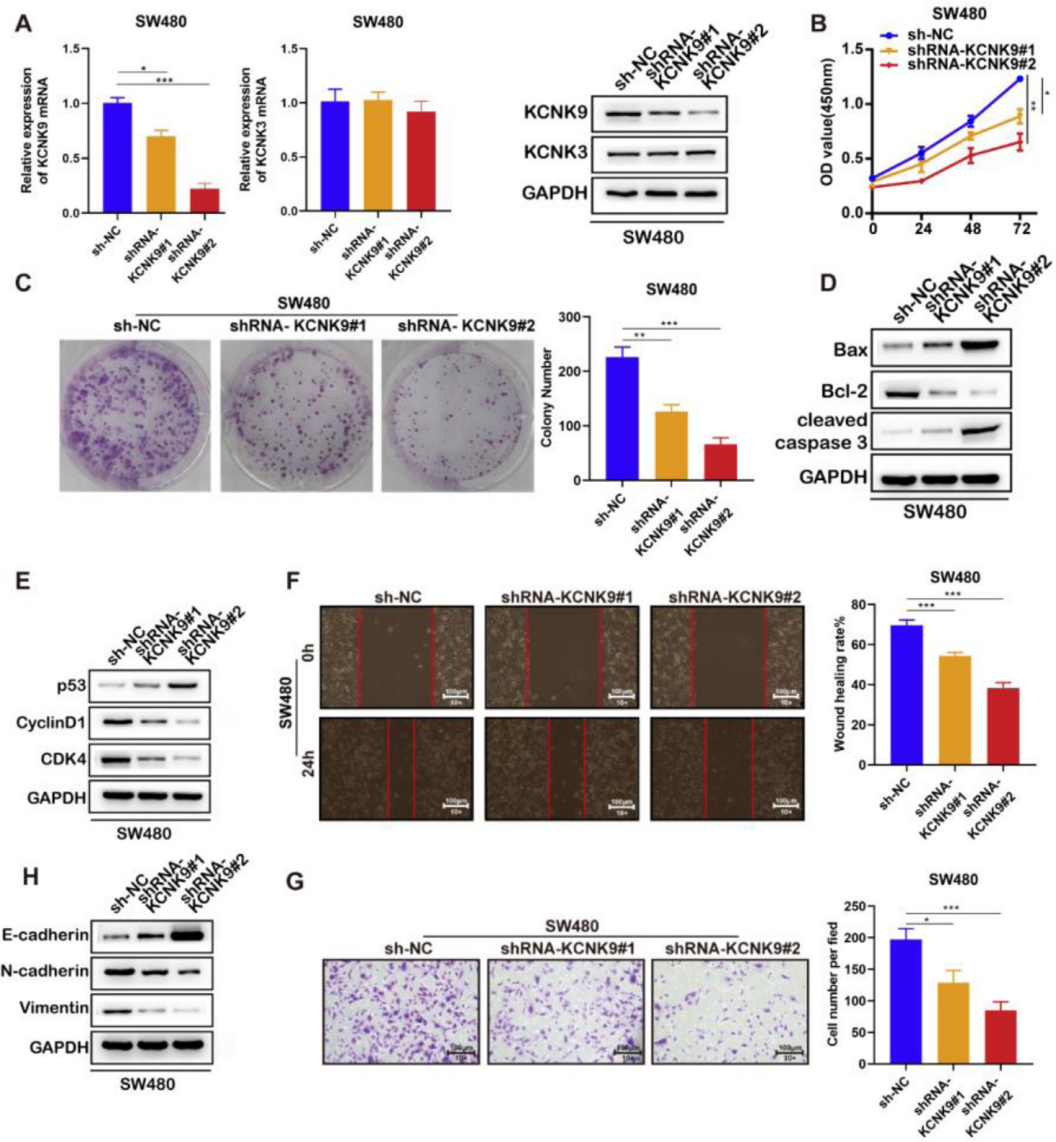
Knockdown of KCNK9 inhibited the malignant phenotype of colon cancer cell line SW480. (A) KCNK9 expression was downregulated and KCNK3 remained unchanged after shRNA application; data were presented as mean ± SD; p-values were calculated using the Student's *t*-test. (B) CCK-8 assay showed decreased cell viability after silencing of KCNK9; Data were presented as mean ± SD; p-values were calculated using the Student's *t*-test. (C) Colony formation assay showed reduced cell proliferation after silencing of KCNK9; data were presented as mean ± SD; p-values were calculated using the Student's *t*-test. (D) Expression levels of Bax, Bcl-2, caspase-3, cleaved caspase-3, PARP, and cleaved PARP were altered after silencing of KCNK9. (E) Expression levels of p53, CyclinD1, CDK6, and CDK4 were altered after silencing of KCNK9. (F) Wound healing assay showed decreased migration after silencing of KCNK9; data were presented as mean ± SD; p-values were calculated using the Student's *t*-test. (G) Transwell assay showed declined cell invasion after silencing of KCNK9; data were presented as mean ± SD; p-values were calculated using the Student's *t*-test. (H) Expression levels of E-cadherin, N-cadherin, and vimentin were altered after silencing of KCNK9.

Next, two KCNK9 knockdown cell lines of SW480 and their negative control cell line were constructed by the same short hairpin RNA (Fig. 4A). Then, the CCK-8 experiment (Fig. 4B), cell clone assay (Fig. 4C), detection of cell apoptosis markers (Fig. 4D), detection of cell cycle markers (Fig. 4E), wound healing assay (Fig. 4F), detection of EMT markers (Fig. 4H), and cell invasion assay (Fig. 4G) were performed, and the results showed a similar outcome after KCNK9 downregulation.

### High expression of KCNK9 was associated with Wnt/β-catenin signaling pathway

**Figure 5:**
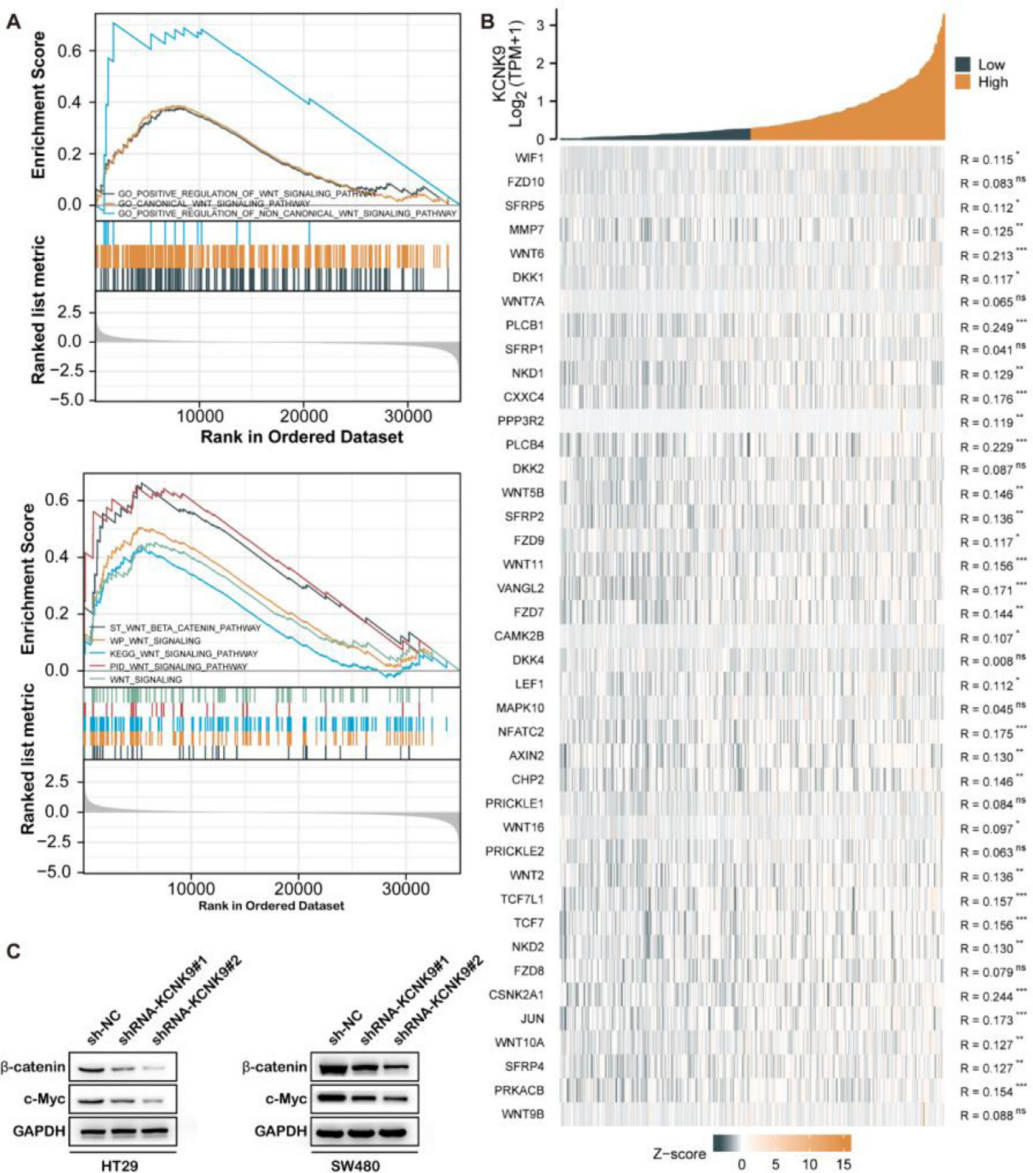
KCNK9 expression was associated with Wnt/β-catenin signaling pathway. (A) Genes in high KCNK9 expression group were enriched into Wnt signaling pathway; (B) The KCNK9 expression level was positively correlated with Wnt signaling pathway; (C) Expression levels of β-catenin and c-Myc after silencing of KCNK9 in HT29 and SW480 cell lines.

For the purpose of finding the KCNK9-related pathway, the GSEA was employed using transcriptome data of colon cancer samples from the Gene Expression Omnibus (GEO) database. Gene Ontology (GO) enrichment analysis showed that high KCNK9 expression samples were enriched in the “Wnt signaling pathway” (Fig. 5A upper). In addition, the results of the Kyoto Encyclopedia of Genes and Genomes (KEGG) pathway analysis indicated that the “Wnt/beta-catenin signaling pathway” was enriched in high KCNK9 expression samples (Fig. 5A). Moreover, it was found that KCNK9 expression was positively correlated with the Wnt signaling pathway-related genes (Fig 5B). Then, the expression levels of β-catenin and its target gene c-Myc in HT29 and SW480 knockdown cell lines were detected. The results showed that the expression levels of β-catenin and c-Myc were downregulated after the interference of KCNK9, indicating that KCNK9 expression was associated with the Wnt signaling pathway (Fig. 5C).

### Genistein inhibited the malignant phenotype of colon cancer cell lines and the activity of the Wnt/β-catenin signaling pathway through KCNK9

**Figure 6:**
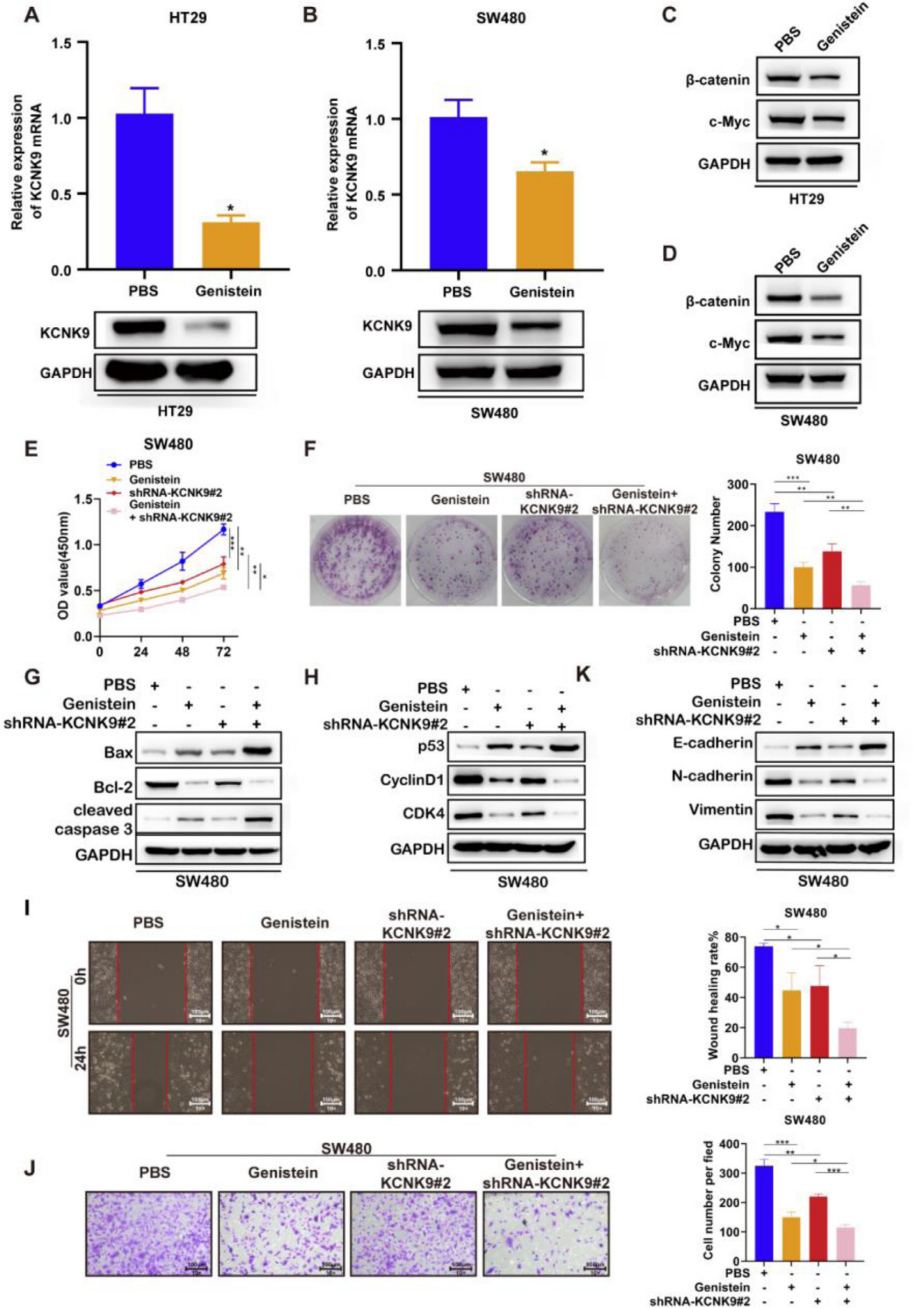
Genistein inhibited KCNK9 expression and Wnt/β-catenin signaling pathway, and genistein application and silencing of KCNK9 could inhibit cell proliferation, promote cell apoptosis, induce cell cycle arrest, decrease cell migration, and restrain EMT in SW480 colon cancer cell line. (A) KCNK9 expression was downregulated in HT29 cell line after administration of genistein; data were presented as mean ± SD; p-values were calculated using the Student's *t*-test. (B) KCNK9 expression was downregulated in the SW480 cell line after administration of genistein; data were presented as mean ± SD; p-values were calculated using the Student's *t*-test. (C) The expression levels of β-catenin and c-Myc were downregulated in the HT29 cell line after administration of genistein. (D) The expression levels of β-catenin and c-Myc were reduced in the SW480 cell line after administration of genistein. (E) CCK-8 assay showed decreased cell viability after genistein application and silencing of KCNK9; data were presented as mean ± SD; p-values were calculated using the Student's *t*-test. (F) Colony formation assay showed reduced cell proliferation after genistein application and silencing of KCNK9; data were presented as mean ± SD; p-values were calculated using the Student's *t*-test. G. Expression levels of Bax, Bcl-2, caspase-3, cleaved caspase-3, PARP, and cleaved PARP were altered after genistein application and silencing of KCNK9; (H) Expression levels of p53, CyclinD1, CDK6, and CDK4 were altered after genistein application and silencing of KCNK9. (I) Wound healing assay showed decreased migration after genistein application and silencing of KCNK9; data were presented as mean ± SD; p-values were calculated using the Student's *t*-test. (J) Transwell assay showed declined cell invasion ability after genistein application and silencing of KCNK9; data were presented as mean ± SD; p-values were calculated using the Student's *t*-test. (K) Expression levels of E-cadherin, N-cadherin, and vimentin were altered after genistein application and silencing of KCNK9.

A previous study revealed that genistein could inhibit the KCNK9 expression level in uterine tissues of female mice and attenuate the Wnt signaling pathway. In order to verify the function of genistein in colon cancer, genistein was used to incubate HT-29 and SW480 cell lines for 48h. The results of RT-qPCR and Western blotting showed that genistein could significantly reduce the KCNK9 expression level in colon cancer cells (Figs. 6A, B). In addition, the expression levels of β-catenin and c-Myc were detected, and it was found that the KCNK9 expression level decreased after genistein treatment. Genistein could also inhibit the Wnt signaling pathway (Figs. 6C, D). Then, KCNK9 knockdown cell lines and control cell lines of SW480 were used to incubate with genistein or PBS. CCK8 experiment showed that genistein treatment could inhibit the viability of colon cancer cells compared with PBS treatment in the control group. Moreover, the knockdown of KCNK9 could further enhance the inhibitory effect of genistein on the viability of colon cancer cells (Fig. 6E). Similar results were revealed by the colony formation assay (Fig. 6F). The expression levels of apoptosis-related proteins were detected, and it was found that genistein treatment could induce apoptosis, while knockdown of KCNK9 could further promote apoptosis (Fig. 6G). The detection of the expression levels of cell cycle-associated proteins showed that genistein could cause cell cycle arrest; moreover, KCNK9 downregulation could further inhibit cell cycle arrest (Fig. 6H). Wound healing assay revealed that genistein reduced the migration capacity of colon tumor cells (Fig. 6I). Cell invasion experiment showed that genistein could inhibit cell invasion capacity, and knockdown of KCNK9 could further inhibit cell invasion (Fig. 6J). The changes in expression levels of E-cadherin, N-cadherin, and vimentin showed a lower EMT level of genistein-incubated cells, and knockdown of KCNK9 could promote this inhibitory effect (Fig. 6K).

### Genistein inhibited liver metastasis of colon cancer cells

**Figure 7:**
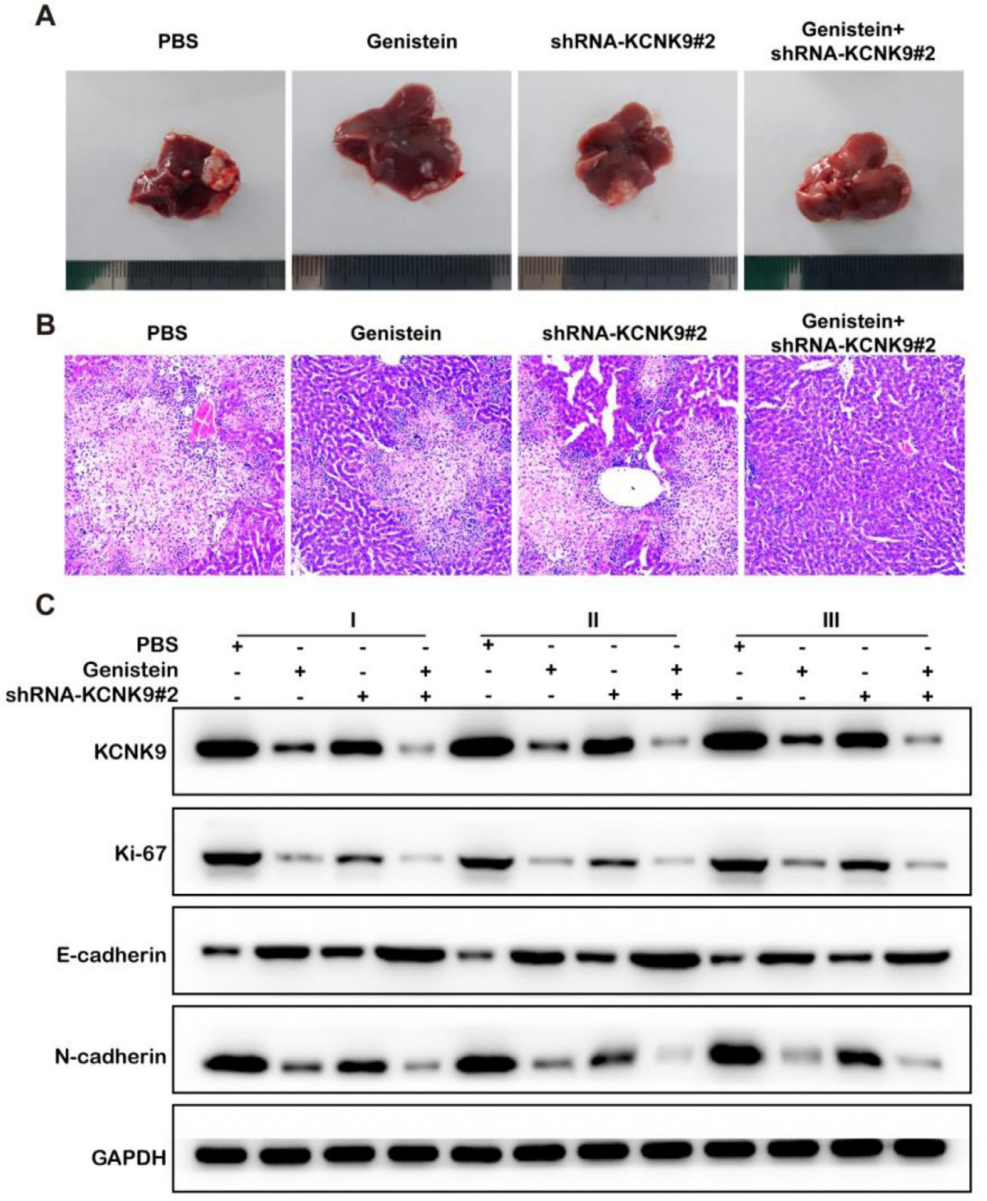
Genistein inhibited the liver metastasis of colon cancer. (A and B) Representative images of metastatic livers and the metastatic nodes that were examined by HE staining (200 ×). (C). Expression levels of KCNK9, Ki-67, E-cadherin, and N-cadherin in hepatic metastatic nodules.

A mouse model of liver metastasis of colon cancer cells was established using the KCNK9 knockdown cell line and control cell line of SW480. Male nude mice were divided into four groups to receive KCNK9 knockdown cells or control cells by intrasplenic cell injection, and they received either PBS or genistein intragastric administration afterward. The liver samples of mice were collected 6-weeks after the spleen injection. H&E staining was applied to detect liver metastatic nodules in mice. It was found that the liver metastatic nodules of KCNK9-knockdown cell lines were smaller than those of control cell lines; in addition, genistein administration could further inhibit the growth of the metastatic nodules (Figs. 7A, B). In order to verify the inhibitory effect of genistein, KCNK9, the proliferation marker Ki67, and the EMT markers, E-cadherin and N-cadherin, in liver metastatic nodules were detected by IHC. It was revealed that genistein administration could suppress KCNK9 expression, reduce cell proliferation, and decrease EMT expression in metastatic nodules. Knockdown of KCNK9 could further promote the inhibitory effect of genistein (Fig. 7C).

## Discussion

KCNK family members are involved in carcinogenesis in numerous types of carcinoma,^9^^,^^24^, ^25^, ^26^ including gastric cancer^27^ and ovarian cancer.^28^ KCNK9 is a member of the KCNK family and is overexpressed in malignancies,^6^^,^^29^, ^30^, ^31^ indicating its cancer-promoting function. Pei et al. demonstrated that G95E point mutation of KCNK9 not only suppressed KCNK9 potassium channel activity but also decreased its oncogenic abilities.^9^ Additionally, the mRNA and protein levels of KCNK9 were altered, and KCNK9 could be used as a prognostic and diagnostic marker for HCC.^7^ In colorectal cancer, mRNA and protein levels of KCNK9 were overexpressed^6^; however, little is known about the role of KCNK9 in colon cancer. The present study highlighted the cancer-promoting effect of KCNK9 through *in vitro* experiments. An elevated KCNK9 expression level in patients with colon cancer was detected. Moreover, the KCNK9 expression level was higher in the subgroup of a tumor with higher malignancy, which indicated that KCNK9 expression was associated with the malignant degree of colon cancer. At the same time, the high KCNK9 expression level was associated with the poorer prognosis of colon cancer patients, suggesting that KCNK9 may act as a protooncogene in colon cancer. In order to verify this hypothesis, the authors constructed two KCNK9 knockdown cell lines of SW480 and HT-29 using shRNA. *In vitro* experiments in both cell lines proclaimed that the knockdown of KCNK9 inhibited the malignant phenotype of colon cancer cell lines. Descendent proliferation level, suspended cell cycle, increased apoptosis, and decreased EMT appeared in KCNK9-knocked down cell lines compared with their vehicles. Bioinformatics analysis revealed that KCNK9 expression was related to Wnt/β-catenin signaling pathway, and experiments showed that it could be inhibited by the downregulation of KCNK9.

Recent studies have demonstrated that genistein could be a promising antitumor drug in preclinical and clinical trials. *In vitro* studies using Huh-7,^32^ Hep3B,^33^ and HepG2^34^ HCC cell lines supported that genistein could be used as a therapeutic candidate against HCC. Genistein could not only affect cell apoptosis and cell cycle but also exert anti-invasive and antimetastatic effects on HCC.^35^ Previous research revealed that genistein could restrain the migration of prostate cancer cell lines^36^ and inhibit the migration of HCC cell lines.^37^ Genistein could also induce cell apoptosis by regulating Akt and nuclear factor-kappa B (NF-κB) signaling pathways.^38^ In addition, genistein is known to lower cyclin D1 expression levels in different types of cancer.^39^^,^^40^ The cell cycle arrest at the G2/M phase could be induced by genistein, thereby decreasing the expression levels of CDK4 and Cyclin D1 in human salivary adenoid cystic carcinoma cell lines.^41^ A wide range of *in vitro* studies have shown that genistein exerted an antitumor activity against colorectal cancer, and its underlying mechanism has been widely studied. In colon cancer, genistein could inhibit the expression level of estrogen receptor-β and mediate the inhibition of human colon carcinoma cell line DLD-1.^42^ Additionally, genistein decreased the protein level of insulin-like growth factor-I receptor (IGF-IR) and efficiently suppressed colon cancer cell proliferation in a concentration-dependent manner by attenuating the activity of the PI3K/Akt signaling pathway.^43^ The results of the present study demonstrated a new antitumor mechanism of genistein that application of genistein downregulated KCNK9 expression and suppressed the malignant phenotype of colon carcinoma by suppressing the Wnt/β-catenin signaling pathway *in vitro*. Additionally, *in vivo* studies indicated that genistein could restrain the liver metastasis of colon cancer and inhibit cell proliferation and the EMT process. The present findings are consistent with the results of a previous study.

## Conclusions

The present study also has some limitations, and the conclusions should be further verified by large-scale studies using data collected from multiple hospitals and databases. In addition, the inhibitory effects of genistein on colon cancer development were only examined by *in vitro* experiments, thus, animal models should be established in the next studies to verify whether genistein can inhibit the occurrence and metastasis of colon cancer. Additionally, KCNK9 could inhibit the Wnt/β-catenin signaling pathway, while the underlying mechanism remains unclear, and the interacting compounds should be clarified. Furthermore, it is pivotal to investigate the possible methods to increase the activity of genistein and testify to the efficacy of the combined therapy of genistein and other clinically used drugs. Despite these limitations, this study demonstrated that genistein could lower KCNK9 expression and the subsequent activation of the Wnt/β-catenin signaling pathway to suppress colon cancer. Inhibition of the Wnt/β-catenin signaling pathway may be one of the promising approaches to treating cancer and increasing the efficacy of chemotherapy. Thus, genistein may help treat colon cancer and show therapeutic benefits.

## Authors’ contributions

Yuan Cheng and Xuping Zhang conceived the study and designed the experiments. Yi Tang, Yiming Tan and Juan Li contributed to the data collection, performed the data analysis, and interpreted the results. Yuan Cheng wrote the manuscript. Xuping Zhang contributed to the critical revision of the article. All authors read and approved the final manuscript.

## Funding

This study was financially supported by Hospital Science and Technology Fund (Grant n° 21YY01 and 20ZYTS01).

## Conflicts of interest

The authors declare no conflicts of interest.

## Appendix Supplementary materials

Image, application 1
