# Supplementary material for: KCNK9 mediates the inhibitory effects of genistein on hepatic metastasis from colon cancer
Source: Clinics (Sao Paulo). 2023 Mar 9;78:100141. doi: 10.1016/j.clinsp.2022.100141 (PMC10019991; doi:10.1016/j.clinsp.2022.100141)
Supplement: Supplementary file 1 [file mmc1.docx]

**CLINICS-D-22-00198 – Supplementary Material**

**Supplementary Table 1** Sequences of shRNA against specific targets**.**

| **shRNA- KCNK9#1** | 5’-3’ | CCTCTTCCCATCGCCTATTAG |
| --- | --- | --- |
| **shRNA- KCNK9#2** | 5’-3’ | GATCTCACCAAGCACATTAAA |

**Supplementary Table 2** Sequences of PCR primers used in this study.

| **KCNK9** | 5’-3’ | ATCAGCAGCGAGGACTACC |
| --- | --- | --- |
|  | 5’-3’ | CGGCGAATTTCCACTGGAC |
| **KCNK3** | 5’-3’ | CTACGAGCACTGGACCTTCTT |
|  | 5’-3’ | CGTAAGGATGTAGACGAAGCTGA |
| **GAPDH** | Forward (5’-3’) | CTCACCGGATGCACCAATGTT |
|  | Reverse (5’-3’) | CGCGTTGCTCACAATGTTCAT |
